# Association of glucocorticoid doses and emotional health in lupus low disease activity state (LLDAS): a cross-sectional study

**DOI:** 10.1186/s13075-021-02466-2

**Published:** 2021-03-10

**Authors:** Yoshia Miyawaki, Sayaka Shimizu, Yusuke Ogawa, Ken-ei Sada, Yu Katayama, Yosuke Asano, Keigo Hayashi, Yuriko Yamamura, Sumie Hiramatsu-Asano, Keiji Ohashi, Michiko Morishita, Haruki Watanabe, Mariko Takano-Narazaki, Yoshinori Matsumoto, Nobuyuki Yajima, Ryusuke Yoshimi, Yasuhiro Shimojima, Shigeru Ohno, Hiroshi Kajiyama, Kunihiro Ichinose, Shuzo Sato, Michio Fujiwara, Hajime Yamazaki, Yosuke Yamamoto, Jun Wada, Shunichi Fukuhara

**Affiliations:** 1 Department of Healthcare Epidemiology, School of Public Health in the Graduate School of Medicine, Kyoto University, Yoshida Konoe-cho, Sakyo-ku, Kyoto, Japan; 2 Department of Nephrology, Rheumatology, Endocrinology and Metabolism, Okayama University Graduate School of Medicine, Dentistry and Pharmaceutical Sciences, 2-5-1 Shikata-cho, Kita-ku, Okayama, Japan; 3 Institute for Health Outcome & Process Evaluation Research (i-Hope International), Akinono-cho 513, Nakagyo-ku, Kyoto City , Kyoto, Japan; 4 Section of Clinical Epidemiology, Department of Community Medicine, Graduate School of Medicine, Kyoto University, Yoshida Konoe-cho, Sakyo-ku, Kyoto, Japan; 5 Division of Rheumatology, Department of Internal Medicine, Showa University School of Medicine, 1-5-8 Hatanodai, Shinagawa-ku, Tokyo, Japan; 6 Center for Innovative Research for Communities and Clinical Excellence, Fukushima Medical University, 1 Hikarigaoka, Fukushima, Japan; 7 Department of Stem Cell and Immune Regulation, Yokohama City University Graduate School of Medicine, 3-9 Fukuura, Kanazawa-ku, Yokohama, Japan; 8 Department of Medicine (Neurology and Rheumatology), Shinshu University School of Medicine, 3-1-1 Asahi, Matsumoto, Japan; 9 Center for Rheumatic Diseases, Yokohama City University Medical Center, 4-57 Urafune-cho, Minami-ku, Yokohama, Japan; 10 Department of Rheumatology and Applied Immunology, Faculty of Medicine, Saitama Medical University, 38 Morohongo, Moroyama-machi, Iruma-gun, Saitama, Japan; 11 Department of Immunology and Rheumatology, Division of Advanced Preventive Medical Sciences, Nagasaki University Graduate School of Biomedical Sciences, 1-7-1 Sakamoto, Nagasaki, Japan; 12 Department of Rheumatology, Fukushima Medical University School of Medicine, 1 Hikarigaoka, Fukushima, Japan; 13 Department of Rheumatology, Yokohama Rosai Hospital, 3211 Kozukue-cho, Kohoku-ku, Yokohama, Japan; 14 Shirakawa STAR for General Medicine, Fukushima Medical University, 1 Hikarigaoka, Fukushima, Japan; 15 Department of Health Policy and Management, Johns Hopkins Bloomberg School of Public Health (JHSPH), Baltimore, Maryland USA

**Keywords:** Systemic lupus erythematosus, Glucocorticoid, Emotional health, Patient-reported outcome, Depression, Anxiety, Cross-sectional study

## Abstract

**Background:**

While survival of systemic lupus erythematosus (SLE) patients has improved substantially, problems remain in the management of their emotional health. Medium to high-dose glucocorticoid doses are known to worsen emotional health; the effect is unclear among patients receiving relatively low-dose glucocorticoids. This study aims to investigate the association between low glucocorticoid doses and emotional health in lupus low disease activity state (LLDAS).

**Methods:**

This cross-sectional study drew on data from SLE patients in 10 Japanese institutions. The participants were adult patients with SLE duration of ≥ 1 year who met LLDAS criteria at the study visit from April 2018 through September 2019. The exposure was the daily glucocorticoid dose (mg oral prednisolone). The outcome was the emotional health score of the lupus patient-reported outcome scale (range: 0 to 100). Multiple linear regression analysis was performed with adjustment for confounders including disease-related damage, activity, and psychotropic drug use.

**Results:**

Of 192 patients enrolled, 175 were included in the analysis. Their characteristics were as follows: female, 89.7%; median age, 47 years (interquartile range (IQR): 37.0, 61.0). Median glucocorticoid dose was 4.0 mg (IQR 2.0, 5.0), and median emotional health score 79.2 (IQR 58.3, 91.7). Multiple linear regression analysis showed daily glucocorticoid doses to be associated with worse emotional health (*β* coefficient = − 2.54 [95% confidence interval − 4.48 to − 0.60], *P* = 0.01).

**Conclusions:**

Daily glucocorticoid doses were inversely associated with emotional health among SLE patients in LLDAS. Further studies are needed to determine whether glucocorticoid tapering leads to clinically significant improvements in emotional health.

**Supplementary Information:**

The online version contains supplementary material available at 10.1186/s13075-021-02466-2.

## Background

Systemic lupus erythematosus (SLE) is a chronic, progressive autoimmune disease that frequently affects young women and can cause damage to organs such as the kidney, heart, lungs, and central nervous system. In SLE patients with high disease activity, irreversible organ damage is likely to occur and the 10-year survival was 50% to 60% until the early 1960s [1]. However, with the development of therapeutic agents and better treatment strategies, survival rates and renal survival estimates 10 years after onset have reached approximately 90% [2]. Yet while survival has improved substantially, prolonged disease duration has a significant impact on patients’ health-related quality of life [3, 4].

One explanation of low quality of life in SLE patients is the high prevalence of anxiety or depressive symptoms [5], which is as high as 40% [6] compared with 4–19% in the general population [7]. In patients with SLE, disease activity contributes to the occurrence of depression and anxiety [8, 9]. In addition, the use of glucocorticoids, which are important therapeutic agents even now, has been known to worsen mental health due to weight gain and cosmetic side effects [10]. Neuropsychiatric symptoms due to the use of glucocorticoids are widely known, whereas no significant association between such impairments of emotional health and glucocorticoid doses has been found in previous studies on quality of life in SLE patients [3, 11]. One study suggested that disease activity could be a potential effect-modifier of the association between emotional health and glucocorticoid doses [11]. To date, there are no reports on the association of emotional health with the limited doses of glucocorticoids used for SLE patients with low disease activity.

In recent years, criteria for lupus low disease activity state (LLDAS) as a therapeutic target for SLE patients have been proposed [12]. The achievement of LLDAS on yearly assessments was negatively associated with the risk of damage accrual [13], compared with those with unstable disease activity, whose control of disease activity should be prioritized. In the present study, therefore, we aimed to evaluate emotional health in SLE patients in the low disease activity state and to assess the association between emotional health and the prescribed daily dose of oral glucocorticoid.

## Methods

### Study design and setting

This was a cross-sectional study using data captured by an ongoing multidisciplinary cohort study (the Lupus registry of Nationwide institutions (LUNA)), which was established in 2016 to investigate the association between clinical manifestations, socioeconomic background, and outcomes in SLE patients reported from 10 Japanese institutions. LUNA contains data on patients aged 20 years or older diagnosed according to the revised 1997 American College of Rheumatology (ACR) criteria [14] for classification of SLE; in LUNA approximately 900 cases, 1.5% of SLE patients in Japan have been registered.

### Data collection

This study was performed using information derived from self-administered questionnaires completed by registered patients or from electronic medical records from April 2018 through September 2019. Data collection was performed annually for each patient and if more than one survey was conducted during the study, only the earliest recorded data were used.

### Study population

Patients registered in LUNA were eligible for this study if all three of the following criteria were met: (1) at least 1 year since SLE onset, (2) history of oral glucocorticoid use, and (3) meeting the definition of LLDAS [12]. LLDAS attainment requires that all four of the following criteria be met: (1) SLE Disease Activity Index 2000 (SLEDAI-2 K [15], scale 0–105) ≤ 4; (2) no active symptoms of major organ involvement (renal, central nervous system, cardiopulmonary, vasculitis, or fever), and no hemolytic anemia or gastrointestinal activity; (3) physician global assessment (PGA, scale 0–3) ≤ 1; and (4) current prednisolone (or equivalent) dose ≤ 7.5 mg daily and well-tolerated standard maintenance doses of immunosuppressive drugs.

### Exposure

The exposure was the prescribed daily amount of oral glucocorticoid used at the time of the survey (mg of oral prednisolone per day or its equivalent). If the patient’s prescribed glucocorticoid dose was unknown, it was handled as missing.

### Outcome measures

The outcome measure was the emotional health domain score out of 12 domains in the Japanese version of lupus patient-reported outcome (LupusPRO) [16, 17]. The emotional health domain consists of these six items: worries about lupus’s impact on future, worries about losing income, anxiety, depression, concern that lupus (or its treatment) may lead to more health problems, and concern that lupus-related health problems will last a long time; each item has a five-point scale with “none of the time,” “a little of the time,” “some of the time,” “most of the time,” “all of the time,” and “not applicable” (recorded as 0 for scoring) [17] used to assess anxiety and depression caused by SLE. The emotional health domain of Japanese LupusPRO was verified for reliability (Cronbach’s alpha coefficient 0.88, test-retest reliability 0.75), convergent validity (Spearman’s correlation coefficient 0.42) using Short Form-12 (SF-12) Mental Health, and for construct validity using confirmatory factor analysis. The emotional health score of the Japanese version of LupusPRO ranges from 0 to 100; a higher score indicates less frequent presence of symptoms [16].

### Potential confounders

Potential confounders based on findings from previous studies [5, 18, 19] and from the clinical perspective of the rheumatologist were the following seven variables: (1) age at the time of the survey, (2) sex, (3) disease duration since SLE diagnosis, (4) damage assessed using the Systemic Lupus International Collaborating Clinics (SLICC)/American College of Rheumatology (ACR) Damage Index [1], (5) disease activity measured using the SLEDAI-2 K score, (6) current use of psychotropics (hypnotics, mood stabilizers, anxiolytics, antidepressants, antipsychotics), and (7) presence of hydroxychloroquine and 8) the number of immunosuppressants.

### Statistical analyses

The descriptive statistics were expressed as the median and interquartile range for continuous variables and as *n* (%) for categorical variables on patients who were divided into four groups by 2.5 mg increments in glucocorticoid dose.

Complete case analysis was performed, excluding patients with missing variables in exposure, outcome, or potential confounders. In the primary analysis, we used a simple regression model to estimate a regression coefficient with a 95% confidence interval (95% CI) between exposures as independent variables and outcome variable. Subsequently, we also conducted a multiple linear regression (ordinary least squares regression) with complete cases to assess the associations between the exposure and the outcome variable after adjusting for potential confounders. The minimal important difference (MID) in health-related quality of life measures is generally interpreted using half a standard deviation (SD) [20] when an anchor-based approach is unavailable, and we used, therefore, 0.5 SD of the emotional health score in the primary analysis population to interpret the magnitude of emotional health impact per 1 mg of glucocorticoid. We visually evaluated using a residual quantile-quantile (Q-Q) plot, a histogram of residuals for detecting violation of the normality assumption, and a residual vs. fitted values plot for detecting non-linearity and unequal error variances.

In the sensitivity analysis, we used multiple imputation to handle the uncertainty caused by missing values of potential confounders on the assumption of missing at random. All variables in the primary analysis were used to create 20 complete data sets in the imputation process. The complete data sets were analyzed by the same procedures for the primary analysis before combining the results from these analyses using Rubin’s rules.

### Statistical significance level and software

All statistical testing was 2-sided; a *p* value of < 0.05 was considered statistically significant. Statistical analyses in this study were performed using the Statistical Package of Stata, version 15.1 (StataCorp, College Station, TX, USA).

## Results

### Patient characteristics

**Fig. 1:**
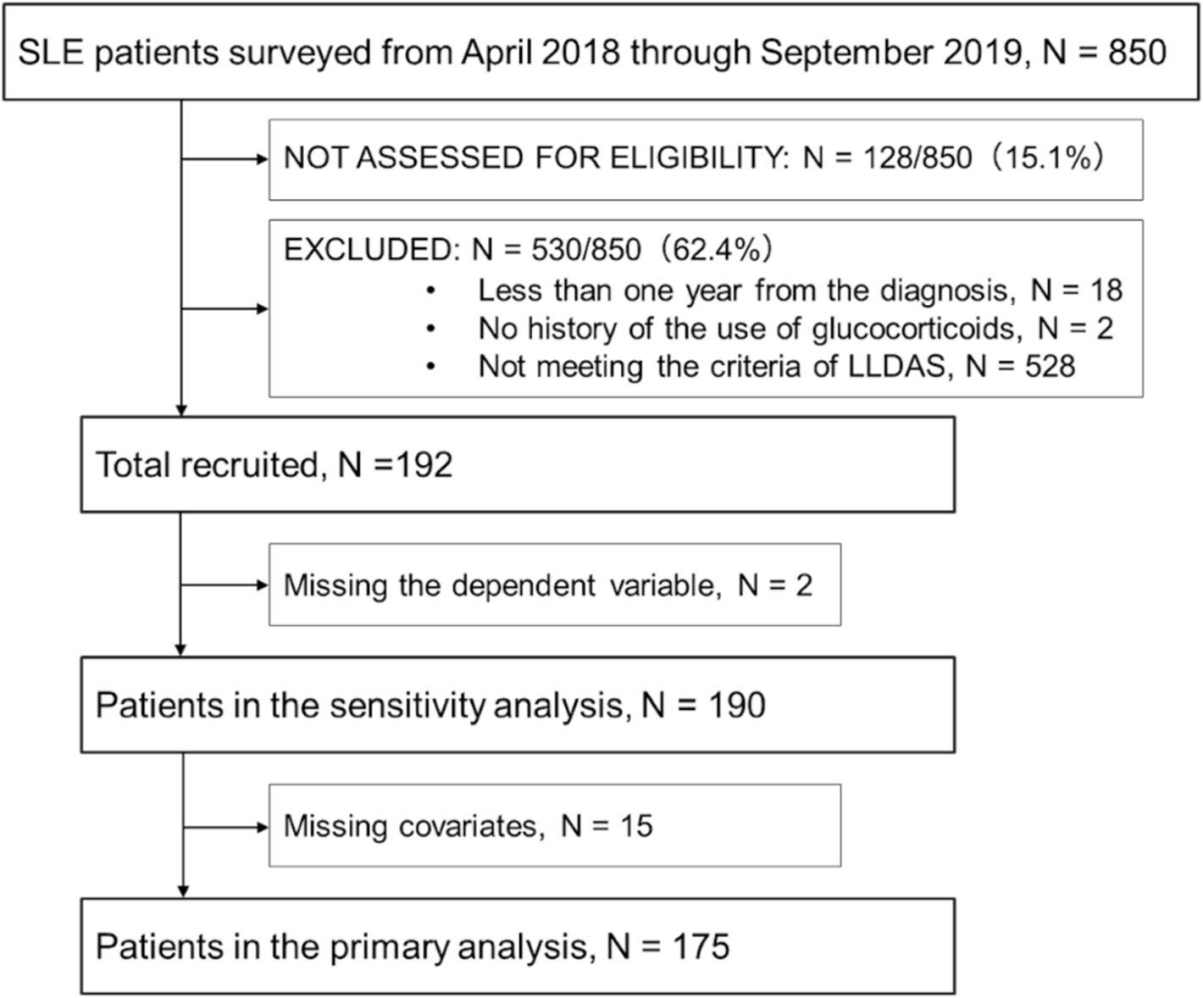
Flow diagram of screening process in the present study. LLDAS, lupus low disease activity state

Of the 850 patients surveyed between April 1, 2018, and August 31, 2019, a total of 192 patients met the inclusion criteria. The eligibility of 128 individuals could not be determined due to missing values, and almost all ineligible patients (528/530 [99.6%]) were ineligible due to failure to achieve LLDAS. We also excluded from the primary analysis the patients with missing values of outcome or with potential confounders. Figure 1 shows the flow of the screening process, and Table 1 shows the patients’ baseline characteristics in the primary analysis.

**Table 1:** Baseline characteristics

	All patients	Glucocorticoid dose (by 2.5 mg/day increments)			
		0 mg/day	> 0 mg/day ≤ 2.5 mg/day	> 2.5 mg/day ≤ 5 mg/day	> 5 mg/day ≤ 7.5 mg/day
Variables	*N* = 175	*N* = 29	*N* = 24	*N* = 92	*N* = 30
Glucocorticoid, median IQR, mg/day	4.0 [2.0–5.0]	0.0 [0.0–0.0]	1.8 [1.0–2.0]	5.0 [4.0–5.0]	6.0 [6.0–7.0]
Age, median IQR, years	47.0 [37.0–61.0]	44.0 [35.0–61.0]	49.0 [38.0–64.5]	47.5 [36.0–59.0]	47.0 [39.0–58.0]
Sex, female, *n* (%)	157 (89.7)	24 (82.8)	22 (91.7)	83 (90.2)	28 (93.3)
Damage index (SDI), median IQR, point	0.0 [0.0–1.0]	0.0 [0.0–0.0]	0.0 [0.0–1.0]	0.0 [0.0–1.0]	0.5 [0.0–1.0]
Time since SLE diagnosis, median IQR, year	12.0 [6.0–19.0]	10.0 [5.0–16.0]	11.0 [6.5–19.0]	12.0 [7.0–20.0]	15.0 [7.0–22.0]
Disease activity index (SLEDAI-2 K), median IQR, point	2.0 [0.0–4.0]	2.0 [1.0–4.0]	2.0 [0.5–4.0]	2.0 [0.0–4.0]	2.0 [0.0–2.0]
Hydroxychloroquine, *n* (%)	46 (26.3)	3 (10.3)	5 (20.8)	24 (26.1)	14 (46.7)
Number of Immunosuppressants					
1, *n* (%)	78 (44.6)	7 (24.1)	6 (25.0)	50 (54.3)	15 (50.0)
2, *n* (%)	15 (8.6)	3 (10.3)	2 (8.3)	5 (5.4)	5 (16.7)
Immunosuppressants					
Cyclophosphamide, *n* (%)	0 (0.0)	0 (0.0)	0 (0.0)	0 (0.0)	0 (0.0)
Mycophenolate mofetil, *n* (%)	15 (8.6)	2 (6.9)	2 (8.3)	7 (7.6)	4 (13.3)
Mizoribine, *n* (%)	12 (6.9)	1 (3.4)	3 (12.5)	6 (6.5)	2 (6.7)
Methotrexate, *n* (%)	3 (1.7)	1 (3.4)	0 (0.0)	1 (1.1)	1 (3.3)
Azathioprine, *n* (%)	15 (8.6)	0 (0.0)	1 (4.2)	11 (12.0)	3 (10.0)
Tacrolimus, *n* (%)	53 (30.3)	8 (27.6)	3 (12.5)	28 (30.4)	14 (46.7)
Cyclosporine, *n* (%)	10 (5.7)	1 (3.4)	1 (4.2)	7 (7.6)	1 (3.3)
Antipsychotics, *n* (%)	10 (5.7)	0 (0.0)	2 (8.3)	5 (5.4)	3 (10.0)
History of diagnosis					
Neuropsychiatric lupus, *n* (%)	6 (3.4)	2 (6.9)	1 (4.2)	2 (2.2)	1 (3.3)
Lupus nephritis, *n* (%)	38 (21.7)	6 (20.7)	3 (12.5)	23 (25.0)	6 (20.0)

Note: Values are presented as the median and numbers in brackets indicate interquartile ranges for continuous variables. Categorical variables are presented as the number and (in parentheses) the percentage of patients divided into four groups by 2.5 mg increments in glucocorticoid dose

*IQR* interquartile range, *SDI* Systemic Lupus International Collaborating Clinics (SLICC)/American College of Rheumatology (ACR) Damage Index, *SLEDAI-2 K* Systemic Lupus Erythematosus Disease Activity Index 2000

The median age was 47.0 (interquartile range (IQR) 37.0, 61.0) years and 157 (89.7%) were female. Nearly half of this cohort was between 20 and 45 years old. The median time since the diagnosis of SLE was 12.0 (IQR 6.0, 19.0) years, the median Systemic Lupus International Collaborating Clinics (SLICC)/American College of Rheumatology (ACR) Damage Index (SDI) was 0 (IQR 0.0, 1.0), and 106 (60.6%) patients had an SDI of 0. The median SLEDAI-2 K was 2.0 (IQR 0.0, 4.0), and 55 (31.4%) patients had a SLEDAI-2 K of 0. The median glucocorticoid dose (exposure) was 4.5 (IQR 2.0, 5.0) mg daily. Both the characteristics of disease activity and the dose of glucocorticoid reflected clinical quiescence in this study population. Patients were taking SLE-related medications, including glucocorticoid, 146 patients (83.4%); hydroxychloroquine, 46 (26.3%); cyclophosphamide, 0 (0%); mycophenolate mofetil, 15 (8.6%); mizoribine, 12 (6.9%); methotrexate, 3 (1.7%); azathioprine, 15 (8.6%); tacrolimus, 53 (30.3%); cyclosporin, 10 (5.7%); and belimumab, 1 (0.6%). The lower the glucocorticoid dose, the less hydroxychloroquine was used.

### Emotional health

**Fig. 2:**
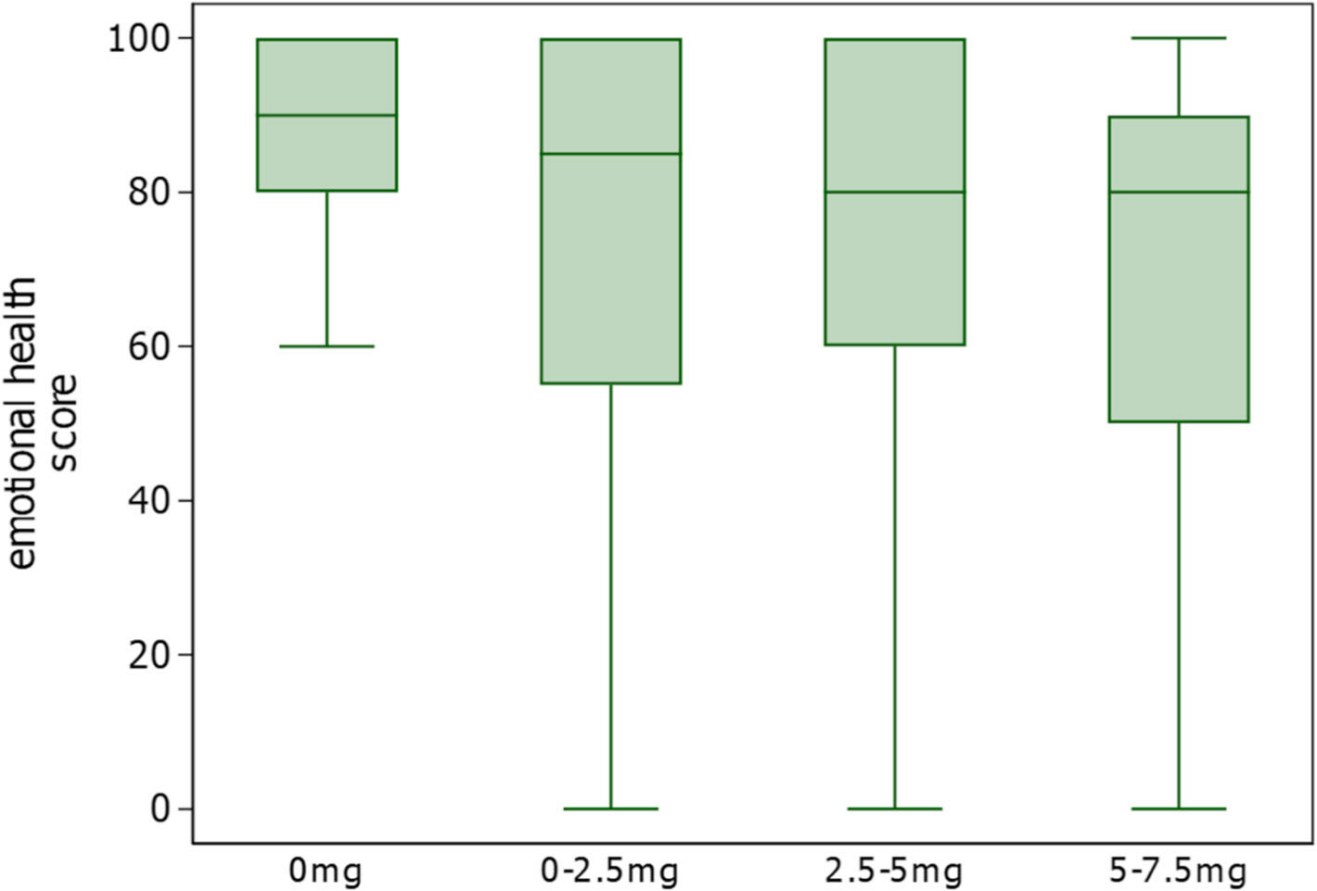
Distribution of emotional health scores according to the dose of glucocorticoid. We used a box-and-whiskers plot to describe the scores on the emotional health domain of Japanese LupusPRO ranging from 0 to 100 (vertical axis) in each dose category of glucocorticoid. LupusPRO, lupus patient-reported outcome

Figure 2 shows the distribution of emotional health scores of LupusPRO for each dose of glucocorticoid. The median emotional health score in the primary analysis population was 79.2 (IQR 58.3, 91.7), with an average of 71.5 (SD 27.3).

### Primary analysis

The results of simple regression analysis (crude) and multiple regression analysis (adjusted) are shown in Table 2. In the adjusted model, the partial regression coefficient *β* = − 2.54 (95% CI − 4.48 to − 0.60, *P* = 0.01) indicated a significant association between the glucocorticoid dose and poorer emotional health. The difference in emotional health score (converted using the SD) per 1 mg of the glucocorticoid dose was − 0.09 SD (− 0.16 SD to − 0.02 SD). Residual diagnostics visually confirmed standard regression assumptions.

**Table 2:** Primary analysis: association between the glucocorticoid dose and emotional health (complete case analysis)

	Crude		Adjusted	
Variables	*β* coefficient [95%CI]	*P* value	*β* coefficient [95%CI]	*P* value
Glucocorticoid, mg/day (per additional mg/day)	− 2.12 [− 3.99, − 0.26]	0.03	− 2.54 [− 4.48, − 0.60]	0.01
Age, years (per 1 year older)			− 0.20 [− 0.55, 0.16]	0.28
Sex, female (vs. male)			11.81 [− 2.10, 25.72]	0.10
Damage index (SDI), point (per additional point)			− 0.90 [− 4.36, 2.55]	0.61
Time since diagnosis of SLE, year (per additional year)			0.30 [− 0.15, 0.75]	0.19
Disease activity index (SLEDAI-2 K), point (per additional point)			− 0.77 [− 3.37, 1.83]	0.56
Hydroxychloroquine (vs. absent)			0.56 [−8.96, 10.07]	0.91
Antipsychotics (vs. absent)			− 1.29 [− 18.88, 16.30]	0.89
Number of immunosuppressants (per additional count)			5.00 [− 1.42, 11.42]	0.13

Note: Covariates: Crude: unadjusted. Adjusted: adjusted for age at the time of the survey, sex, time since diagnosis of SLE, damage index, disease activity index, current use of psychotropics (hypnotics, mood stabilizers, anxiolytics, antidepressants, antipsychotics) and the presence of the immunomodulator hydroxychloroquine

*SDI* Systemic Lupus International Collaborating Clinics (SLICC)/American College of Rheumatology (ACR) Damage Index, *SLEDAI-2 K* Systemic Lupus Erythematosus Disease Activity Index 2000, *SLE* systemic lupus erythematosus. 95% confidence intervals (CI) in brackets

### Sensitivity analysis

After multiple imputation of missing values, the partial regression coefficient *β* = − 2.73 (95% CI − 4.63 to − 0.84, *P* < 0.01) indicated a negative association between the glucocorticoid dose and emotional health, similar to the findings of the primary analysis.

## Discussion

Using data from LUNA, a multicenter prospective Japanese cohort, the present study brought to light the distribution of emotional health in patients who met the criteria for lupus low disease activity state (LLDAS). On the emotional health domain of the LupusPRO scale, we found a negative association with the dose of glucocorticoid, which suggested that the lower the glucocorticoid dose, the better a patient’s emotional health.

The results of this study showed that even a low dose of prednisolone-equivalent of 7.5 mg daily or lower was associated with altered emotional health such as anxiety or depression. Although cases of onset even at doses as low as 7.5 mg or less have been reported [21], no studies have focused on the association between such impairments of emotional health as anxiety and depression and low-dose prednisolone in patients with SLE. Neuropsychiatric symptoms due to the use of glucocorticoid are widely known [22], and the association between emotional health and daily doses of glucocorticoid (prednisolone or equivalent 7.5 mg or more) has been demonstrated in other diseases such as chronic obstructive pulmonary disease [23] and adrenal insufficiency [24], but the association was not clear in SLE patients. A previous study on SLE [3] might have adjusted insufficiently such confounding factors as age, sex, disease duration, damage, current use of psychotropics, and hydroxychloroquine compared to this study. Yet another previous study, which evaluated the longitudinal association between mental health scores and glucocorticoid doses, used comprehensive measures of health outcome rather than disease-specific status [11]; thus it may have been unable to detect differences in SLE patient-specific emotional health scores. LupusPRO may have been the key to detecting more disease-specific anxiety and depression caused by disease-specific factors in comparison with using a comprehensive scale [11]. Items in emotional health in LupusPRO assess not only anxiety and depression, but also other emotional aspects such as worries about lupus’s impact on the future, worries about losing income, and concern that lupus (or its treatment) may lead to more health problems. These studies also included patients with a broader spectrum of disease activity, which could be an important effect modifier, and these may help to explain the findings of the present study.

This study has several limitations. First, we were unable to select variables for socioeconomic status as covariates because of the high proportion of missing values of such variables as marital status and educational attainment. A previous analysis of the quality of life of SLE patients reported that the influence of these socioeconomic status variables is not associated with quality of life [25]. The residual confounding effects of such unmeasured variables as history of mental disorder, relapses of disease activity occurring within the past year, duration of LLDAS, past treatment experience, and cumulative doses of glucocorticoid from the onset have not been taken into account. Past disease activity and cumulative glucocorticoid doses are associated with SDI [26]. Both the number of concomitant immunosuppressants and SDI were adjusted to minimize the impact of residual confounding factors in this study, but this attempt may have been insufficient. Second, most patients in this study were followed in a tertiary care practice, and therefore, these results may not be applicable to patients with LLDAS in other settings. Patients who generally go to a secondary or tertiary care institution have a clinically severe course; thus, we should consider that the population in this study actually tends to have poorer emotional health scores than SLE patients with LLDAS as a whole. Third, the cross-sectional nature of this study may cause reverse causality, which may have resulted in attribution of lower doses of glucocorticoids to patients with good emotional health.

This study’s strengths are first that it was conducted using clinical data collected from a prospective multicenter cohort study as an objective measure of the features and outcomes of SLE patients. Second, in order to scrutinize the association between emotional health and the glucocorticoid dose, considering effect modification by disease activity, we separated SLE patients into subgroups using the criteria for LLDAS proposed in 2016. Third, our study had more statistical power with a greater sample size than that of previous studies on quality of life of SLE patients, which had sample sizes of about 100 participants.

The aggressive use of hydroxychloroquine and other immunosuppressive drugs during the periods of remission-maintenance might make further reductions in glucocorticoid doses possible, thereby permitting improvements in emotional health that exceed the minimal important difference. Recent clinical practice guidelines state that the glucocorticoid dose can be tapered as long as the disease activity is stable; however, a lack of consensus exists regarding the tapering schedules and maintenance dose of glucocorticoid [27]. Although no data on minimal important differences [28] in emotional health of LupusPRO have been reported and this cross-sectional study is limited to mentioning minimal important differences in individuals, every 1 mg increase in the dose of glucocorticoid is equivalent to a 0.09 SD lower emotional health score, indicating that a 0.5 SD difference, generally considered the minimal important difference [20], corresponds to a 5–6 mg per day dose of glucocorticoid. Further studies are needed to determine whether reducing the glucocorticoid dose is indeed possible, whether glucocorticoid tapering is associated with the achievement of the minimal important difference in emotional health, and whether similar results are confirmed using other disease-specific measures such as LupusQOL, for which the minimal important difference has already been established.

## Conclusion

The daily dose of glucocorticoid was negatively associated with emotional health among systemic lupus erythematosus patients in lupus low disease activity state. Further longitudinal studies are needed to determine whether glucocorticoid tapering is associated with clinically significant improvements based on LupusPRO’s minimal important difference in emotional health in patients in lupus low disease activity state.

## Supplementary Information

**Additional file 1.**

## Data Availability

The dataset analyzed in this paper is available from the corresponding author on reasonable request.

## Abbreviations

ACR: American College of Rheumatology
CI: Confidence interval
IQR: Interquartile range
LLDAS: Lupus low disease activity state
LUNA: Lupus registry of nationwide institutions
LupusPRO: Lupus patient-reported outcome
MID: Minimal important difference
PGA: Physician global assessment
Q-Q: Quantile-quantile
SD: Standard deviation
SF-12: Short Form-12
SLE: Systemic lupus erythematosus
SLEDAI-2 K: SLE disease activity index 2000
SLICC: Systemic Lupus International Collaborating Clinics
