# Supplementary material for: Association of glucocorticoid doses and emotional health in lupus low disease activity state (LLDAS): a cross-sectional study
Source: Arthritis Res Ther. 2021 Mar 10;23:79. doi: 10.1186/s13075-021-02466-2 (PMC7944592; doi:10.1186/s13075-021-02466-2)
Supplement: Supplementary file 1 — Additional file 1. [file 13075_2021_2466_MOESM1_ESM.docx]

Supplementary table 1 Baseline characteristics

|  | | All patients |  | Glucocorticoid dose (by 2.5 mg/day increments) | | | | | | |  |
| --- | --- | --- | --- | --- | --- | --- | --- | --- | --- | --- | --- |
|  | |  |  | 0 mg/day |  | > 0 mg/day  <=2.5 mg/day |  | > 2.5 mg/day  <=5 mg/day |  | > 5 mg/day <=7.5 mg/day |  |
| Variables | | N = 175 |  | N = 29 |  | N = 24 |  | N = 92 |  | N = 30 |  |
| Antimalarials and immunosuppressants | |  |  |  |  |  |  |  |  |  |  |
|  | Neither hydroxychloroquine or immunosuppressants, n (%) | 59  (33.7) |  | 16  (55.2) |  | 13  (54.2) |  | 25  (27.2) |  | 5  (16.7) |  |
|  | Both hydroxychloroquine and immunosuppressants, n (%) | 23  (13.1) |  | 0  (0.0) |  | 2  (8.3) |  | 12  (13.0) |  | 9  (30.0) |  |
|  | Hydroxychloroquine only, n (%) | 23  (13.1) |  | 3  (10.3) |  | 3  (12.5) |  | 12  (13.0) |  | 5  (16.7) |  |
|  | Immunosuppressants only, n (%) | 70  (40.0) |  | 10  (34.5) |  | 6  (25.0) |  | 43  (46.7) |  | 11  (36.7) |  |

Note: Categorical variables are presented as the number and (in parentheses) the percentage of patients divided into four groups by 2.5 mg increments in glucocorticoid dose.
